# Disaster management of the psychological impact of the COVID-19 pandemic

**DOI:** 10.1186/s12245-021-00342-z

**Published:** 2021-03-24

**Authors:** Mohamud Sheek-Hussein, Fikri M. Abu-Zidan, Emmanuel Stip

**Affiliations:** 1 Institute of Public Health, College of Medicine and Health Sciences, UAE University, Al-Ain, United Arab Emirates; 2 Department of Surgery, College of Medicine and Health Sciences, UAE University, Al-Ain, United Arab Emirates; 3 Department of Psychiatry and Behavioral Sciences, College of Medicine and Health Sciences, UAE University, Al-Ain, United Arab Emirates

**Keywords:** COVID-19, New Corona virus, Distress, Psychiatry, Mental stress, Domestic violence

## Abstract

**Background:**

The COVID-19 pandemic has exposed a suboptimal response to this threatening global disaster, including the response to the psychological impact. Both the economic hardship and the continuous media coverage of alarming news have exacerbated this effect which also includes increased domestic violence.

**Aim:**

To address this important aspect of disaster management and provide recommendations on how to mitigate these effects.

**Methods:**

This is a narrative review written by three experts in community medicine, disaster medicine and psychiatry reflecting the interdisciplinary approach in managing disasters. Selected important papers, personal published papers, PUBMED articles and media news related to the disaster management of the psychological effects of COVID-19 pandemic were collected over the last year, critically appraised and used in writing this manuscript.

**Results:**

The COVID-19 pandemic causes major *emotional distress*. Lack of effective treatments and availability of the current vaccines for this virus increases the fear of being infected and infecting others. Negative emotions are common and are related to adjustment but may progress in the long term to anxiety, depression, and post-traumatic stress syndrome. The COVID-19 pandemic has a major impact on *mental health*. The most common distress reactions include anxiety, insomnia, perception of insecurity, anger, fear of illness, and risky behaviors. Patients having mental disorders are vulnerable during the pandemic because of (1) somatic vulnerability, (2) cognitive and behavioral vulnerability, (3) psychosocial vulnerability, and (4) disruption to psychiatric care. Psychiatric wards, which are commonly separate from main hospitals, should be included in the disaster management plans. Acute care physicians carry the psychological and ethical impact of difficult triage decisions when ending the support of some patients to save others. A combination of fear and guilt may overcome normal human tolerance levels in vulnerable health workers. The moral injuries can be carried for a long time.

**Conclusions:**

Addressing the psychological effects is an essential component of disaster management of infectious pandemics. This should be implemented through the whole spectrum of disaster management including preparedness, mitigation, response, and recovery.

## Background

Throughout history, the world has struggled to combat the constant threat of emerging viral pandemics. The 1918 influenza pandemic killed around 50 million people globally [1]. Other threatening infectious viral outbreaks have occurred during the last two decades, including SARS-CoV in 2002, H1N1 in 2009, the Middle East respiratory syndrome coronavirus in 2012, Ebola in 2014, and Zika in 2015 [2, 3].

The world changed dramatically during the last century. Transportation methods evolved rapidly, such that now the globe is like a village with short travel times between its parts. This contributed to the swift advance of COVID-19 which spread worldwide, large numbers being infected within the last year. To date, there have been more than a hundred million infected cases and more than two million deaths globally [4].

The current novel coronavirus (COVID-19) pandemic which started in Wuhan City of Hubei Province of China in December 31, 2019 [5] has exposed existing weaknesses in public health infrastructure globally as well as the lack of preparedness and suboptimal response for such pandemic disasters, including for psychological aspects [6–8].

COVID-19 is a highly contagious lower respiratory tract infection which can be transmitted through droplets or by touching surfaces contaminated during coughing or sneezing from both symptomatic and asymptomatic persons [9]. Patients who are highly contagious are called super spreaders. They can quickly infect multiple individuals within a range of 2 m [10, 11]. The symptoms of COVID-19 disease include fever, cough, myalgia, and fatigue, and appear after an incubation period of 2–14 days [11–13]. However, the majority of infected subjects are carriers without clinical symptoms. Over time additional symptoms that are frequently associated with COVID-19 infection have been identified, such as loss of smell and taste (anosmia and dysgeusia) [14].

Swift contact tracing is pivotal for infection control: close contacts of symptomatic patients should be identified as early as possible and isolated. The public were asked not to shake hands, to stay at a distance of 2 m or more from others, and to use face masks to reduce the spread of the infection [11]. Such behavior is unusual and may lead to discomfort and difficulty complying. Commonly, these measures are legally required with strict penalties for infringement of regulations to protect the community.

There is no doubt that the COVID-19 pandemic is a major stressor that is impacting mental health worldwide. The human experiences encountered during the COVID-19 pandemic may be potentially damaging psychologically, physically, socially, and spiritually. They cause a crisis of conscience, which is not limited to developing countries or war-zones [15].

Psychological impact has usually been ignored or has received only limited attention in disaster management plans. This review aims to address the early psychological impact of the COVID-19 pandemic, giving suggestions on how to mitigate these effects on the community, including health care providers, in the current COVID-19 pandemic as an essential component of disaster management.

## Contributory factors for psychological effects of COVID-19 pandemic

### Stay at home orders

The majority of people were ordered to stay at home for long periods and work from there except those having essential jobs such as in food delivery, pharmacies, healthcare work, and jobs in basic social infrastructure. This caused reduced physical activity, which has negative effects on mental health in the community because physical activities directly reduce general negative emotions [16]. This further affects the usual ethical human autonomy of choice. However, ethical considerations are different in disasters, as under such circumstances one should save as many patients as possible even if it affects some personal rights. This is an ethical situation faced by acute care physicians during triage.

The role of prolonged proximity and contact with family members at home during the pandemic while working may stress the employees especially if their work needs time to reach the peak plateau of concentration. This interruption may reduce the productivity and increase the stress especially in obsessional high intellectual persons. This may demand a change in the environment which cannot be easily fulfilled in the new work-from home life-styles.

The effects of claustrophobia, loneliness, and the need for company as contributing factors for psychological impact of COVID-19 pandemic has to be addressed. Claustrophobia is an anxiety disorder related to specific places in which a patient cannot stay. The confinement situation of COVID-19 pandemic is not really an “enclosed space” but more related to the reduced daily activities. Both claustrophobia and a stress-related anxiety produce real “panic attacks” with a feeling of imminent death. In claustrophobia, the anxiety is localized leaving the other psycho-behavioral fields free. Respiratory protective devices (RPDs) may cause respiratory discomfort in panic-prone individuals [17]. Furthermore, anxiety may develop to coronaphobia which is associated with avoidance of public places and events [18, 19].

### Modern media effects

The style of modern life has changed with more people opting to live alone without family members, and being connected more to the media and computers. This is different from the small cities and villages of former times when direct contact and communication between people was essential. These major changes in human behavior made such individuals more susceptible to the psychological effects of the COVID-19 pandemic.

The COVID-19 pandemic is unique. Advanced technology makes it possible for individuals to update themselves with continuous alarming news. When waking up in the morning, the first thing individuals do is to search for the latest numbers of infected patients and deaths. The continuous rise in numbers increases fear and anxiety. Similarly, before individuals go to sleep, they check the numbers again. This pattern has consequences. News on COVID-19 continues to make the headlines, with daily counts of the number of deaths. This will be a source of stress and anxiety for those who are intensely exposed to it, especially those with underlying disease. The increased number of deaths was strongly correlated with reduced sleep, which in turn significantly increased negative emotions, stress, and anxiety [16].

### Economic impact

The COVID-19 pandemic is having a major impact on the global economy. It has caused the most severe economic recession of the last hundred years, with tremendous damage to jobs and savings, especially for young workers. It is anticipated that gross domestic product (GDP) will be reduced by 9.1% in a single wave COVID-19 pandemic and 11.5% in a double wave pandemic [20]. The stock market fell by 35% by April 2020. There was a major fall in oil prices due to reduced demand because of the lockdown. Brent crude fell to 21 US dollars per barrel. The number of flights dropped dramatically worldwide, with more than 100 countries closing their airports [21]. Low-wage laborers continued to work under difficult conditions to support their families. African Americans, as an example, have high COVID-19 mortality compared with Caucasians [22]. It is possible that economic hardship increased the exposure of low-income workers to the virus. These low-wage workers have been struggling between the worry of not supporting their families and fear of infection.

The measures to slow the transmission and spread of the coronavirus infection, ranging from limiting contact by “social distancing” to full curfew, had significant negative effects on the economy. This has increased the stress especially on low-income manual workers who depend mainly on their daily wages to support their families. Low-income countries may not even be able to afford free protective and preventive measures including PPEs and vaccination. This will further increase the stress on the mental health of individuals including the health care providers.

## Spectrum of psychological effects

### Emotional distress

Early diagnosis and detection, isolation, and quarantine are important public health measures to combat the spread of COVID-19 despite the development of effective vaccines against the virus. It is still a long way before the world population is properly vaccinated because the rate of vaccination is slow relative to the spread of the virus. This makes the public frightened of infection and fearful to approach or take care of their infected relatives. Currently, supportive care and prevention of complications are the main pillars of COVID-19 management. Consequently, major emphasis is put on measures to reduce person-to-person transmission so as to control and prevent spread within the community, particularly for the most susceptible population. This has included restricting social gatherings, travel, transportation, and recreational activities. The working environment has changed. Employees have been encouraged to work online from home if possible. Those above 60 years old or having co-morbidities have been asked to stay at home. Schools have been closed and children stayed with their families unable to leave the home [3, 11].

An infectious pandemic often causes emotional distress, even in those not directly exposed to it. The Canadian experience during the SARS epidemic showed that patients with SARS reported “fear, loneliness, boredom and anger, and they worried about the effects of quarantine and contagion on family members and friends” [23]. Healthcare personnel expressed fear of contagion and of infecting their loved ones and colleagues. Uncertainty and stigmatization were prominent in both staff and patients [24–28].

Healthcare workers who are in constant close contact with COVID-19 patients in healthcare institutions, especially in front-line areas (like the emergency departments and critical care units) and those with pre-existent psychological disorders are more susceptible to the psychological effects of COVID-19 pandemic. Adverse mental health consequences for frontline healthcare workers have been reported before in relation to the previous SARS outbreaks [29].

### Mental distress

**Fig. 1:**
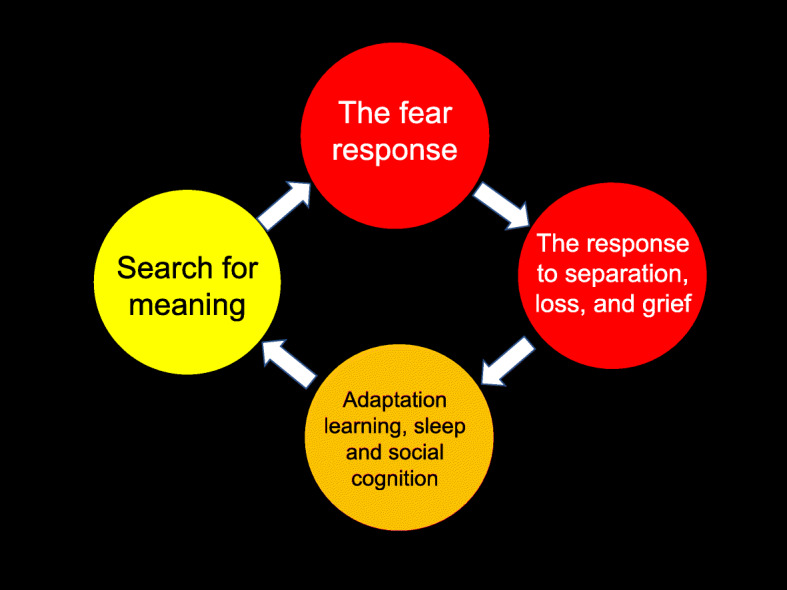
Universal psychological responses after disasters are modulated by the interaction between four responses: (1) the fear response; (2) the response to separation, loss, and grief; (3) adaptation learning, sleep, and social cognition; and finally (4) search for meaning

Because of the quarantine and self-isolation, which are unlikely to be lifted in the near future in many countries, the prevalence of psycho-traumatic symptoms among the public will potentially increase. According to Lupien [30], stressors can be classified under the acronym CINE, namely weak **C**ontrol; “**I**mprevisible” or unpredictability; **N**ovelty; and the threat to **E**go. *Weak control* is the feeling that you have little or no control over the situation in which you find yourself. *Unpredictability* is experiencing unexpected events, or inability to know what will happen to you. *Novelty* is expecting something new to happen that you have never experienced before. Lastly, Ego threat includes a situation posing a perceived direct threat to your ego. Further, universal psychological responses after disasters are modulated by the interaction between four responses: (1) the fear response; (2) the response to separation, loss, and grief; (3) adaptation learning, sleep, and social cognition; and finally, (4) search for meaning (Fig. 1).

### Domestic violence

Domestic violence (DV) increases during natural disasters [31–33]. For example, there was a fourfold increase in partner DV among displaced women following the Hurricane Katrina disaster [34]. It is therefore logical to extrapolate that DV would increase during the COVID-19 epidemic. It is predicted that there will be an increase of more than 30 million in the number of victims of gender-based violence worldwide during COVID-19 lockdown [35].

Stay at home orders are not favorable for victims of DV because they are locked in with their abusers and isolated from their social support networks. These effects are exacerbated by emotional stress and economic hardship. Victims usually accept the risk of staying with their abusers because their abusers control their daily needs while threatening them if they are not obeyed [31, 36, 37]. Furthermore, victims are afraid of COVID-19 infection if they leave their abusive partners [31].

Women and immigrants are more vulnerable to DV. During the COVID-19 pandemic in Europe, there has been a 60% increase in emergency telephone calls from women subjected to DV [35]. Data from US police departments have shown an increase of 10% to 28% in DV in four different states [38]. This could well be an underestimate because many victims might not call for help unless violence has reached a point of intolerable damage [31]. These effects are more profound in US immigrants who may be afraid of deportation if they report abuse [37].

### Effects on health care workers

Health-care workers are under huge physical and mental stress during the current COVID-19 pandemic as they are on the front line fighting COVID-19 [39]. They carry the psychological impact of difficult triage: they may have to decide to stop the support of some to save others, due to lack of ventilators, meanwhile bearing the fear of being infected themselves. Some work with limited personal protective equipment (PPE). A recent study from Italy has shown that strategies to protect surgeons against COVID-19 infection were suboptimal compared with the WHO standards in more than 70% of hospitals, while advanced PPE was adopted for all operations in fewer than 13% of all hospitals [40]. A combination of fear and guilt may overwhelm normal human tolerance levels in vulnerable health workers. This may cause moral injuries that must be borne for a long time; some may show signs of burnout [41].

These effects are more pronounced in acute care physicians, who have to deal with shortages of PPE and ventilators, communicate with stressed families and break bad news to them, while taking care of themselves and their own families. Some have to stay away from their loved ones for extended periods to protect them from infection [42, 43]. Some have died and others have suffered the loss of their own loved ones while on duty. Twenty percent of health-care workers taking care of COVID-19 patients in Italy were infected [44]. A recent report from Italy showed that 28.2% of surgical departments had at least one member infected by COVID-19, while 33.8% had a member quarantined [40]. We know from war experience that witnessing a member of your own unit seriously injured or killed during combat is one of the most important factors associated with war-related post-traumatic stress syndrome [45]. This can be easily extrapolated for health care workers who see their own colleagues get sick and die from COVID-19 before their eyes despite their tremendous efforts to save them.

## Clinical presentation

The COVID-19 pandemic has a major impact on mental health [46]. The most common psychological and behavioral reactions are distress reactions which include anxiety, insomnia, perception of insecurity, anger, increased use of health devices for fear of illness, and behaviors risky for health (increased consumption of alcohol, illicit drugs and tobacco, change in work-life balance, social isolation, increased family conflicts and violent behavior) [7, 47, 48] (Table 1).

**Table 1:** Psychological and behavioral experiences with COVID19 pandemic

● Anxiety, rumination, and panic	
● Confusion and difficulty in concentration	
● Tendency to question too much	
● Irritability	
● Disturbance of sleep and appetite (eating or sleeping too much)	
● Bravery, denial	
● Multiple bereavements	
● Loss of job, feeling of guilt for inability to support family	
● Social withdrawal, loss of leisure, cuts and religious meetings	

The psychological consequences of the pandemic are difficult to predict and will depend on multiple individual and collective factors. These factors include loneliness, prior vulnerability, quarantine duration, resilience, and access to and quality of care. It is natural to experience fear of contamination, helplessness and boredom, temporary sleep disturbances, concern for those close to you, irritability, and feelings of frustration. These negative emotions which are experienced in the current situation are common and related to adjustment. However, such short-term moderate symptoms are likely to progress in the long term to anxiety, depressive and adjustment disorders, addiction, and post-traumatic stress disease (PTSD). Accordingly, they should be monitored, explained, and de-dramatized [49–51].

In this context, some individuals may develop depressive or anxiety disorders or PTSD which require specific treatment. The presence of significant and repetitive symptoms over time, such as panic attacks, increased substance use, persistent insomnia, nightmares, daytime hypervigilance, cognitive complaints, anhedonia, and even suicidal ideation should not be ignored, but diagnosed and treated appropriately. The prevalence of significant symptoms of PTSD 1 month after the onset of the COVID-19 pandemic in the most affected regions reached 7% [52]. A recent meta-analysis using seven databases showed that the combined prevalence of symptoms of PTSD was 33% (0–86), anxiety 28% (21–36), stress 27% (14–43), and depression 22% (13–33). In this quantitative review, the prevalence of psychological symptoms was similar among health workers and the general population (34% (24–44) and 33% (27–40) respectively) [53]..

A recent study on 1257 health care workers from Wuhan China who were on the front line managing COVID-19 patients found that they experienced symptoms of depression (50.4%), anxiety (44.6%), insomnia (34%), and distress (71.5%) [27]. Sadly, some doctors could not cope with the psychological impact and committed suicide [54]. The period following the acute phase of the coronavirus epidemic is the hardest for medical professionals in terms of psychological impact. The long-term impact of COVID-19 during the recovery period is not yet clear. Further research is important to explore these effects [55].

Multiple respiratory viral infections can produce neurological symptoms [56]. For example, SARS-CoV-2 disease may cause anosmia, meningitis, or encephalitis. Coronaviruses are possibly neurotropics. They can enter the brain via the olfactory system causing complex neuropsychiatric symptoms [57, 58]. Their interaction with psychotropic drugs should be considered. COVID-19 may affect the blood levels of certain psychotropic drugs, including antipsychotics such as clozapine, or mood stabilizers such as lithium [59, 60]. Conversely, certain psychotropic drugs such as chlorpromazine may have a beneficial effect on corona virus infection [61].

## Diagnosis and follow up

Health care providers should be educated and trained on the recognition and mitigation of mental health problems related to COVID-19 pandemic and how to respond to them. Psychiatric patients and victims of domestic violence should be followed up, supported, and counseled regularly through video conferences during pandemics.

Virtual telepsychiatry plays an important role during infectious pandemics. It enables treating physicians to communicate directly with those patients who need continuous care in a safe environment. This will also reduce levels of fear in the patients. The outpatient clinic can be quickly transformed into a virtual telepsychiatry unit [62]. Patients are usually highly satisfied with telepsychiatry because they can be followed without the risk of COVID-19 [63].

Telemedicine interventions may ameliorate some of the effects of DV, providing a new helpful communication tool for DV screening. Health care workers should be properly trained in telemedicine communication skills if they want to gain the trust of DV victims while using this method [37].

Although telephone interviews were useful for triage in fever clinics during the COVID-19 pandemic, these were not encouraged for psychiatric interviews. There are some concerns regarding phone consultations, which include lack of confidence in the diagnosis in new patients and the inability to see the facial expression of the patients [64]. Nevertheless, it may be helpful if the diagnosis is already established and there were sufficient previous communications and established trust between the patients and the treating physicians.

## Management

### Patient treatment

Mental health professionals may treat patients having increased emotional distress caused by the effects of the pandemics on them, on their families, or on their community. Health-care providers may need counseling and help if they experience psychological hardship during and after the pandemic.

It is important to stress that managing psychological patients in disasters should have a multi-factorial approach. Patients suffering from mental disorders are vulnerable to the effects of COVID-19 because of four factors: (1) somatic vulnerability; (2) cognitive and behavioral vulnerability; (3) psychosocial vulnerability, which are all related to the patient (Table 2); and finally (4) vulnerability caused by the disruption to psychiatric care during the pandemic. All four of these factors should be properly addressed during disasters.

**Table 2:** Patient-related vulnerability to COVID19 pandemic in mental health population

Vulnerability	Mechanism	Cause of reduced life expectancy
Somatic	• Schizophrenia, bipolar disorder, depression, anxiety disorder, or autism	• Higher risk of pneumococcal infections
	• Cross immuno-genetic vulnerability • Risk factors for severity of COVID-19 infection	• Lower capacity to defend themselves against infections • Comorbidities: cardiovascular, pulmonary pathologies, diabetes, obesity, smoking tobacco
Cognitive and behavioral	• Compliance to follow mitigation and advices and treatment • Delusional interpretation	• Relapse with psychiatric symptoms • Exposition to virus increase • Lower response to treatment
Psychosocial	• Social isolation • Precarious housing • Little solidarity network • Low income • Forensic	• Sub-optimal monitoring of health and mitigation • Vulnerability of prison psychiatric populations

The difficulties of educating psychiatric patients and making them adopt barrier measures and infectious isolation are particularly challenging. Psychiatric patients should be hospitalized if deemed necessary, for example if there was a risk to their life, or if they lack family support. It is important to note that in many countries psychiatric wards are remote from somatic medicine services and medical resuscitation units both geographically and professionally. This should be modified in disaster management plans.

### Monitoring the plasma levels of psychotropic drugs

Most psychotropic drugs should be reduced by 25–50% when patients receive lopinavir or ritonavir [65]. Pneumonia with an intracellular pathogen can be hyponatremic. Some serotonergic antidepressants may also cause hyponatremia [66], and respiratory syndrome could be aggravated by benzodiazepine, carbamates, or opioids. The potential aggravation of respiratory symptoms indicates the need to re-assess the risk-benefit balance of these treatments [67]. Furthermore, COVID-19 medications may interact with psychiatric medications such as pimozide, midazolam, and carbamazepine. Dose titration should be gradual and guided by ECG monitoring if cardiac toxicity was suspected and Qt period was prolonged [68–70].

### Providing adequate resources

Services and resources should be available and sufficient during disasters. They should be tailored according to the stage of the disaster and to the needs of different communities. Plans should assure the availability of PPE, and antidepressant and antipsychotic drugs during the pandemic.

Furthermore, sufficient resources should be available to support DV victims. Comprehensive DV guidelines that can be used during the response and recovery stages of disasters are already available [71]. The effects of the COVID-19 pandemic on DV and the methods to address it should be an integral part of disaster management in all its stages, and should be included in future research on the effects of COVID-19 [36].

## Mitigation and prevention

Practicing normal activities like sports, drawing, cooking, or interacting with the family during the lockdown is highly recommended. The WHO, governments, and research all recommend limiting individual exposure to COVID-19 news through traditional and social media to a maximum of 2 h per day. Only useful information should be received [72]. When a threat is uncertain, it is normal to seek information; nevertheless, overexposure to stressful news heightens the feeling of threat, absorbs considerable time daily, and can even paralyze an individual, and prevent them from adopting protective behaviors and facing life demands [73]. Following the media should stop at least 1 hbefore sleep to reduce insomnia [74, 75]. Healthy individuals are advised to communicate by phone or video meetings instead of emails.

For health professionals, mentally overexposed to the pandemic especially with the second wave, bibliotherapy has become an effective and inexpensive alternative to traditional face-to-face therapy. It is also an alternative to the academic Journal Club which leaves no room for emotions and stress management. Reading at one’s own pace with follow-up sessions by phone, videoconference, or virtual reality allows people with economic, geographic, physical, or mental barriers to benefit from bibliotherapy [76]. In addition, social media such as WhatsApp can be used to promote and maintain social interactions during the social isolation associated with COVID measures [77].

Prevention of COVID-19 infection from reaching the psychiatric wards, which are usually remote from somatic medicine services, should be carefully planned. Infection control policies in psychiatric wards should be regularly monitored. PPE should be available for psychiatric health care providers, with proper training on how to use it in case they need to restrain patients suspected to have COVID-19 disease.

Professional health organizations may consider community behavioral therapy and information management during and after the pandemic so as to ensure that information provided to the public is accurate and authoritative which is very useful for the management of the current pandemic and preparedness for future ones. For example, the qualitative information gathered from dedicated helpline set up have rich information that can be used for this purpose [29].

In addition, psychological first-aid teams in all sectors of society should be created as a preventive measure so that such teams may be activated for disasters, including pandemics, to manage those with early signs of psychological stress.

## Conclusions

Addressing psychological effects is an essential component of the disaster management of infectious pandemics. These effects can be serious and can affect healthy subjects, patients, and health care providers. Appropriate measures should be implemented through the whole spectrum of disaster management including the preparedness, mitigation, response, and recovery.

## Data Availability

There is no additional data available to share with the readers.

## Abbreviations

DV: Domestic violence
ECG: Electrocardiography
GDP: Gross domestic product
PPE: Personal protective equipment
PTSD: Post-traumatic stress disease
RPDs: Respiratory protective devices
WHO: World Health Organization
